# Care of the Ears

**Published:** 1892-07

**Authors:** 


### CARE OF THE EARS.

Much misdirected energy is expended by careful people in the effort to keep clean the innocent orifice of the organ of hearing.

Serious injury often results to the delicate mucous membrane lining the canal of the ears from the pushing of wash-cloths, sponges and the like inside the delicate canal. Nothing should ever be pushed inside the canals of the ears. The cerumen or wax which is normally found there should not be removed until it can be washed away with ordinary washing; this should not include a doubling or twisting of the end of a wash-cloth for the purpose of pushing it inside the auditory canal.

It is common enough to find those who use pins, hair-pins and other hard bodies to remove the normal secretion of the ear from the canal. A physician is the only one who should put into the ear any thing so hard as possibly to injure its delicate structure. If there is any thing abnormal about the quantity or quality of the natural secretion, a physician should be consulted and his advice followed.

No one should attempt himself to treat any supposed or real case of impacted or hardened cerumen. Efforts in this direction have been extremely harmful to the tympanum and delicate bones of the ears. Such attempts have also brought on the dreaded condition—which was before only a supposed one—by massing the cerumen at a narrowed point of the canal.

The upper layer of epithelium of the membrane of the canal has the wonderful property of moving outward toward the opening of the ear, while still continuing part of the membrane. Thus a scab may be seen at one time quite near the drum of the ear, and afterward be found considerably nearer the orifice. In this way the protective wax is pushed gently outward without further assistance.

It is a common fallacy to suppose that any dullness of hearing is beyond the help of the physician, and that, consequently, nothing can be done for it. Slight dullness of hearing is often occasioned by a catarrhal condition of the throat, which dullness gets better or worse as the condition of the membrane of the throat changes. The great majority of cases of deafness, it can be safely said, are not beyond improvement, or at least a checking of the degenerative process.

A discharging, or "running," ear should always have treatment at once.—*The Youth's Companion.*

